# A noninvasive optical approach for assessing chloride extrusion activity of the K–Cl cotransporter KCC2 in neuronal cells

**DOI:** 10.1186/s12868-017-0336-5

**Published:** 2017-01-31

**Authors:** Anastasia Ludwig, Claudio Rivera, Pavel Uvarov

**Affiliations:** 1 Neuroscience Center, University of Helsinki, Helsinki, Finland; 2 INSERM U901, Institut de Neurobiologie de la Méditerranée (INMED), Marseille, France; 3 UMR S901, Aix-Marseille Université, Marseille, France; 4 Department of Anatomy, Faculty of Medicine, University of Helsinki, Helsinki, Finland; 5 École Normale Supérieure, Institut de Biologie de l’ENS (IBENS), INSERM U1024, CNRS 8197, Paris, France; 6 Department of Biosciences and Neuroscience Center, University of Helsinki, Helsinki, Finland

**Keywords:** Genetically encoded chloride sensor, *Slc12a5* gene, KCC2, Inhibition, GABA

## Abstract

**Background:**

Cation-chloride cotransporters (CCCs) are indispensable for maintaining chloride homeostasis in multiple cell types, but K–Cl cotransporter KCC2 is the only CCC member with an exclusively neuronal expression in mammals. KCC2 is critical for rendering fast hyperpolarizing responses of ionotropic γ-aminobutyric acid and glycine receptors in adult neurons, for neuronal migration in the developing central nervous system, and for the formation and maintenance of small dendritic protrusions—dendritic spines. Deficit in KCC2 expression and/or activity is associated with epilepsy and neuropathic pain, and effective strategies are required to search for novel drugs augmenting KCC2 function.

**Results:**

We revised current methods to develop a noninvasive optical approach for assessing KCC2 transport activity using a previously characterized genetically encoded chloride sensor. Our protocol directly assesses dynamics of KCC2-mediated chloride efflux and allows measuring genuine KCC2 activity with good spatial and temporal resolution. As a proof of concept, we used this approach to compare transport activities of the two known KCC2 splice isoforms, KCC2a and KCC2b, in mouse neuronal Neuro-2a cells.

**Conclusions:**

Our noninvasive optical protocol proved to be efficient for assessment of furosemide-sensitive chloride fluxes. Transport activities of the N-terminal splice isoforms KCC2a and KCC2b obtained by the novel approach matched to those reported previously using standard methods for measuring chloride fluxes.

## Background

Cation-chloride cotransporters (CCC) form a protein family comprising nine members, which play an important role in maintaining chloride homeostasis in neuronal, renal, vascular, and other cell types [1]. The only member of the CCC family that shows an exclusively neuronal expression in mammals is the potassium–chloride (K–Cl) cotransporter 2 (KCC2) [2]. KCC2 is indispensable for processes of neuronal migration in developing central nervous system, formation and maintenance of small dendritic protrusions—dendritic spines—in maturing neurons, and for rendering fast hyperpolarizing responses of ionotropic γ-aminobutyric acid (GABA) and glycine receptors in adult neurons [3]. KCC2 deficiency in mature neurons results in elevated levels of intracellular chloride concentration [Cl^−^]_i_ and, as a consequence, in attenuated levels of fast hyperpolarizing GABAergic and glycinergic inhibition [4]. Two protein isoforms KCC2a and KCC2b, which differ only in their most N-terminal parts, are encoded by *Slc12a5* gene [5]. Complete genetic ablation of the *Slc12a5* gene in mice results in severe motor deficits and absence of respiratory rhythm, causing death immediately after birth [6, 7]. Mice with a specific deletion of the KCC2b isoform express only 5–8% of a basal KCC2 protein level, exhibit frequent generalized seizures, and die 2–3 weeks postnatal [8]. In line with this, three recent studies have associated totally five missenses mutations in the human KCC2 gene with cases of epileptic seizures [9–11]. The identified KCC2 mutants demonstrate deficit of intrinsic transport activity and/or impaired plasmalemmal expression that considerably reduced chloride extrusion activity. Neuropathic pain is another severe outcome of the impaired GABAergic and glycinergic signaling in central pain pathways that is accompanied by downregulation of KCC2 expression and/or activity [12]. Development of novel drugs augmenting KCC2 activity and/or expression after neuronal trauma has been declared as a plausible way to treat neuropathic pain [13, 14]. Analysis of chemical compounds by high-throughput screening (HTS) requires reliable and robust methods for assessing KCC2 transporter activity in neuronal cell lines.

Both electrophysiological and non-electrophysiological methods have been used so far to analyze activity of the KCC2 protein and its mutant isoforms, though direct measuring of chloride fluxes in neuronal cells by electrophysiological tools is hampered by the electroneutral nature of the K–Cl cotransport. Yet, several indirect methods allow assessing KCC2 transport activity by exploiting the fact that a reversal potential for inotropic GABA_A_ (E_GABA_) and glycine (E_Gly_) receptors depend on [Cl^−^]_i_. Thus, E_GABA_ in a gramicidin-perforated patch configuration, which does not disturb [Cl^−^]_i_, provides a close estimation for a steady-state [Cl^−^]_i_ level [15]. This approach, however, does not necessarily reflect an actual extrusion activity of KCC2, as in the conditions of low cellular chloride conductance even a relatively weak KCC2 activity may significantly decrease [Cl^−^]_i_. Another approach is to use a whole-cell patch clamp configuration for measuring somatodendritic E_GABA_ gradient in conditions of constant Cl^−^ loading via somatic patch pipette [16]. One possible caveat of this method is that the endogenous KCC2 activity could be significantly distorted by continuous Cl^−^ loading, since SPAK and OSR1 kinases, which have been previously shown to regulate KCC2 activity [17–19], are known to be inhibited by high [Cl^−^]_i_ [20]. One way to avoid this problem is to assess efficiency of neuronal Cl^−^ extrusion by measuring a recovery time constant (τ) of changes in inhibitory postsynaptic potential mediated by GABA_A_ receptors (IPSP_A_) after a brief injection of chloride currents by iontophoresis [21–23]. The short-term character of Cl^−^ injection minimizes an impact on SPAK/OSR1 signaling cascade, thus allowing a genuine KCC2 extrusion activity to be measured by fitting a curve of IPSP_A_ amplitude recovery immediately after the cessation of Cl^−^ injection. This approach has proven to be useful for a pharmacological dissection of different components of chloride conductance in neurons.

Electrophysiological methods described above are labor intensive and time consuming, thus lacking a high efficiency required for HTS applications and for analyzing activity of multiple KCC2 mutants. Moreover, instead of measuring electroneutral KCC2-mediated chloride fluxes directly, these methods provide only indirect correlates of the KCC2 transport activity (steady-state [Cl^−^]_i_, τ, somatodendritic E_GABA_ gradient). Another strategy to quantify K–Cl cotransport is to directly analyze dynamics of [Cl^−^]_i_ changes in response to imposed chloride gradients [24]. One of the approaches to follow [Cl^−^]_i_ dynamics is to measure intracellular accumulation of radioactive chloride (^36^Cl^−^), though a relatively long half-life time of ^36^Cl^−^ isotope (around 300,000 years) implies a substantial radioactive hazard especially in case of HTS applications [25]. Intracellular chloride concentration can also be measured using chloride-sensitive microelectrodes, but such electrodes are difficult for construction and cannot be used for HTS applications. Chemical assays such as silver chloride gravimetry and atomic absorption spectroscopy have been used for measuring [Cl^−^]_i_ previously and are still in use [26]. However, both methods are tedious since they require multiple washing steps and separation of cellular and extracellular solutions. One more strategy to overcome problems described above is to quantify K–Cl cotransport by analyzing dynamics of K^+^ instead of Cl^−^ component. Indeed, in case of various potassium channels and transporters, K^+^ cation can be substituted for its congeners such as radioactive ^86^Rb^+^ [27, 28], nonradioactive ^85^Rb^+^ [29, 30], Tl^+^ [31–33], and NH_4_^+^ [34], thus fluxes of these cations instead of K^+^ can be measured. Although such approaches have been successfully exploited for CCC members in HTS applications in nonneuronal cell lines, their implementation for neuronal cells has so far been limited due to same technical reasons as for the described above Cl-flux methods (poor survival of neuronal cells after multiple washing steps, poor loading and high toxicity of Tl^+^ sensitive dyes, a relatively high amount of cells required for an analysis, health hazard issues in case of radioactive ^86^Rb^+^ isotope). Moreover, neuronal expression of numerous K^+^ channels and transporters impedes an accurate assessment of K^+^-fluxes attributed specifically to KCC2. These methods provide low temporal resolution and lack spatial resolution needed for measuring KCC2 activity in small neuronal compartments—axons, dendrites, and dendritic spines.

To overcome limitations inherent to the electrophysiological and K^+^ flux-related methods, development of a new class of tools—genetically encoded chloride sensors (GECS)—has been commenced a decade and a half ago [35]. At that time, it was found that fluorescent characteristics of green fluorescent protein (GFP) variants depend on the concentration of halide anions [36]. Since then various GECS have been characterized: YFP-H148Q [37] with multiple variants [38] including a membrane-targeted mbYFPQS [39], Clomeleon [40] and its recent modification SuperClomeleon [41], ClopHensor [42] with several modifications [43] including the neuronal variant ClopHensorN [44], as well as Cl-sensor [45] with its glycine receptor (GlyR)-linked variant BioSensor-GlyR [46]. Most of the above-mentioned chloride sensors are fusion proteins comprising a halide-sensitive (YFP, E^2^GFP) and a halide-insensitive (CFP, DsRed, tdTomato) parts connected by a short linker. Such design allows ratiometric measurements to be done in either FRET or non-FRET mode. A major advantage of GECS is that they make possible noninvasive [Cl^−^]_i_ measurements in neuronal somas and compartments (axons, dendritic spines and shafts). Moreover, many cells can be recorded simultaneously providing a high efficiency required for HTS applications [13]. Furthermore, stable cell lines encoding GECS can be easily propagated in quantities required for screening of large libraries of chemical compounds. Transgenic mice expressing YFP [47], Clomeleon [48], and Cl-sensor [49] have also proved to be useful for studying Cl^−^ dynamics in brain slices and in dissociated neuronal cultures.

A major disadvantage of the YFP-based chloride sensors is their relatively high sensitivity to protons [37, 40, 43, 45, 47, 50–52]. Indeed, inaccuracy in determination of [Cl^−^]_i_ depends on the basal [Cl^−^]_i_ and pH_i_ and may reach up to 10 mM [40, 45] at physiologically relevant low [Cl^−^]_i_ [44, 53]. To measure [Cl^−^]_i_ more precisely, independent pH_i_ recording using pH-sensitive dyes or genetically-encoded pH sensors is necessary, especially in the cases of significant pH_i_ fluctuations induced by neuronal activity [54]. This can be accomplished by using ClopHensor—a chloride sensor that allows simultaneous noninvasive [Cl^−^]_i_ and pH_i_ measurements. Even though ClopHensor and its variants [43] provide more accurate [Cl^−^]_i_ measurements [42, 44], usage of these sensors is more technically demanding compared to other GECS for several reasons: (1) three excitation wavelengths instead of two, normally used for other GECS, are needed for acquisition; (2) usage of laser light sources is preferable [42, 44] because two excitation wavelengths for ClopHensor (488-nm pH-dependent E^2^GFP signal and 458-nm pH-independent E^2^GFP signal) are closely set; (3) additional photodiode, which measures laser power fluctuations during acquisition, has to be installed into optic paths to correct fluorescence ratios [42, 44]; and (4) a relatively complex data analysis [35].

In the current study, we describe a protocol for noninvasive measuring of KCC2 transport activity in neuronal cells. We have attempted to combine advantages and to avoid known weaknesses of the previously described methods for assaying KCC2 transport activity. Our approach measures KCC2-mediated Cl^−^ fluxes in a robust way using a previously characterized genetically encoded chloride sensor Cl-sensor. In contrast to ClopHensor and its derivatives, usage of Cl-sensor requires neither expensive laser light sources nor a confocal setup [45, 55, 56] and can be accomplished with a conventional fluorescence microscope [57]. Moreover, instead of measuring steady-state [Cl^−^]_i_ levels—indirect correlate of the KCC2 activity, our protocol directly assesses dynamics of KCC2-mediated Cl^−^ fluxes. This new approach can be used for screening chemical compounds augmenting KCC2 activity as well as for assessing a transport activity of KCC2 mutant variants. As a proof of concept, we use this method to compare transport activities of the two previously characterized KCC2 isoforms, KCC2a and KCC2b [5, 58] in neuronal type Neuro-2a cells.

## Methods

### DNA constructs

**Fig. 1:**
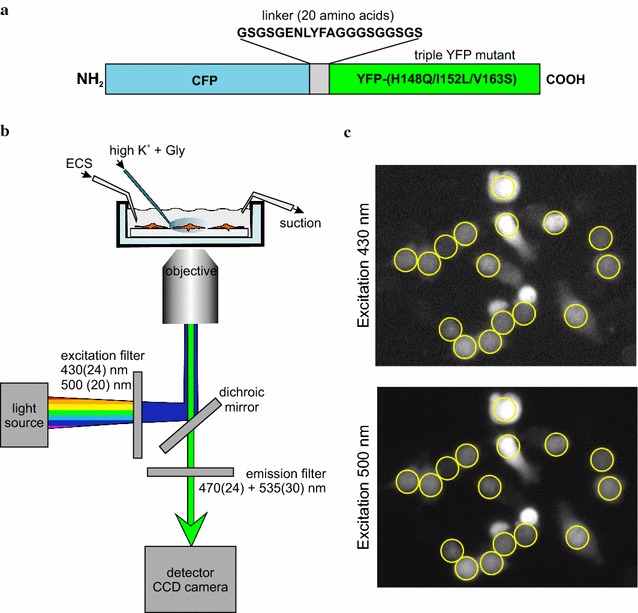
Experimental design for assessing the chloride extrusion activity of the K–Cl cotransporter KCC2 in neuronal cells. **a** Schematic depiction of the Cl-sensor. Cl-sensor consists of a triple YFP mutant fused via a short 20 amino acid linker with the CFP protein. **b** Schematic picture of the inverted fluorescence imaging setup used in the study. Grown on a coverslip, Neuro-2a cells were perfused with extracellular solution (ECS) in the absence and then in the presence of furosemide. Combination of high [K^+^] and glycine (with and without furosemide) was applied via the local perfusion system during the chloride-loading step. Details of the excitation and emission filters are described in the “Methods” section. **c** Representative optic view of Neuro-2a cells consequently excited with the 430 and 500 nm light. Regions of interest (ROIs) used for the subsequent analysis of the emitted fluorescence intensities for this optic view are indicated by *yellow circles*

Cl-sensor consists of the chloride sensing YFP triple mutant YFP-(H148Q/I152L/V163S) connected by a 20 amino acid linker (GSGSGENLYFAGGGSGGSGS) to the chloride-insensitive CFP [45] (Fig. 1a). Cl-sensor in pGW-CMV expression vector (British Biotechnology, Oxford, UK) and human GlyR α1 subunit expression construct were described previously [55, 59]. Expression constructs for KCC2a and KCC2b splice variants of the neuronal KCC2 cotransporter were characterized previously [5, 58]. pcDNA3.1(−) plasmid (Invitrogen) was used for a mock transfection control.

### Cell lines and transfection

**Fig. 2:**
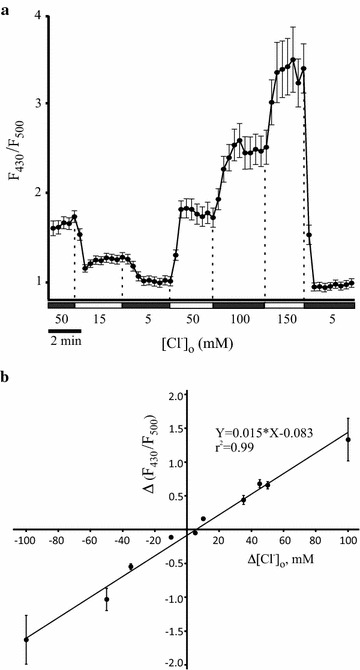
Calibration of the Cl-sensor in Neuro-2a cells. **a** Ratio for intensities of fluorescent signals emitted by Neuro-2a cells expressing Cl-sensor after excitation with 430 nm (F_430_) and 500 nm (F_500_) light. Neuro-2a cells were permeabilized with β-escin and incubated in extracellular solutions containing different Cl^−^ concentrations [Cl^−^]_o_. An example of stepwise [Cl^−^]_o_ changes is shown. Acquisition interval is 20 s. Mean values and the corresponding SEM are shown for 12 cells recorded in the optic field. **b** A linear regression for F_430_/F_500_ changes Δ(F_430_/F_500_) obtained for a broad range of [Cl^−^]_o_ changes (Δ[Cl^−^]_o_) derived from multiple (n = 8) independent experiments similar to one shown in (**a**). Each data point shows how the F_430_/F_500_ ratio changes in response to a change of the extracellular chloride concentration Δ[Cl^−^]_o_. *Error bars* represent SEM. *Error bars* corresponding to −10, +5, and +10 mM data points are too small to be seen on the graph

**Fig. 3:**
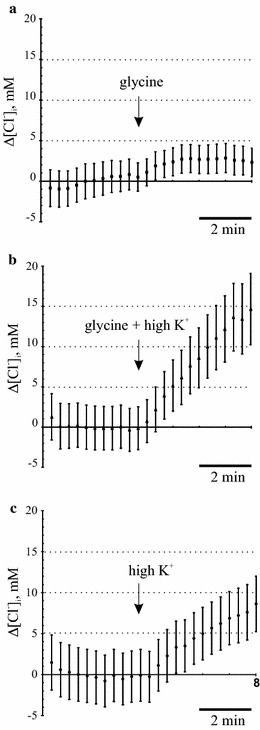
Glycine in combination with high K^+^ provides an efficient chloride loading into Neuro-2a cells exogenously expressing glycine receptors. **a** In the absence of high K^+^ in the extracellular solution (ECS), application of glycine to Neuro-2a cells expressing Gly receptors results only in a minor increase in the intracellular chloride concentration. In non-depolarized Neuro-2a cells, chloride loading is inefficient even in cells with relatively low [Cl^−^]_i_ and may be completely blocked in cells with high [Cl^−^]_i_ levels. Mean values and corresponding SEM are shown for n = 13 recorded cells. **b** Application of glycine in combination with high K^+^ strongly increases the intracellular chloride concentration. Note that in the depolarized conditions opening of the Cl-permeable channels upon glycine application always results in a robust Cl^−^ accumulation. Mean values and corresponding SEM are shown for n = 20 recorded cells. **c** Application of high K^+^ along increases the intracellular chloride concentration at the rate about twice slower compared to the co-application of glycine and high K^+^. Mean values and corresponding SEM are shown for n = 14 recorded cells

**Fig. 4:**
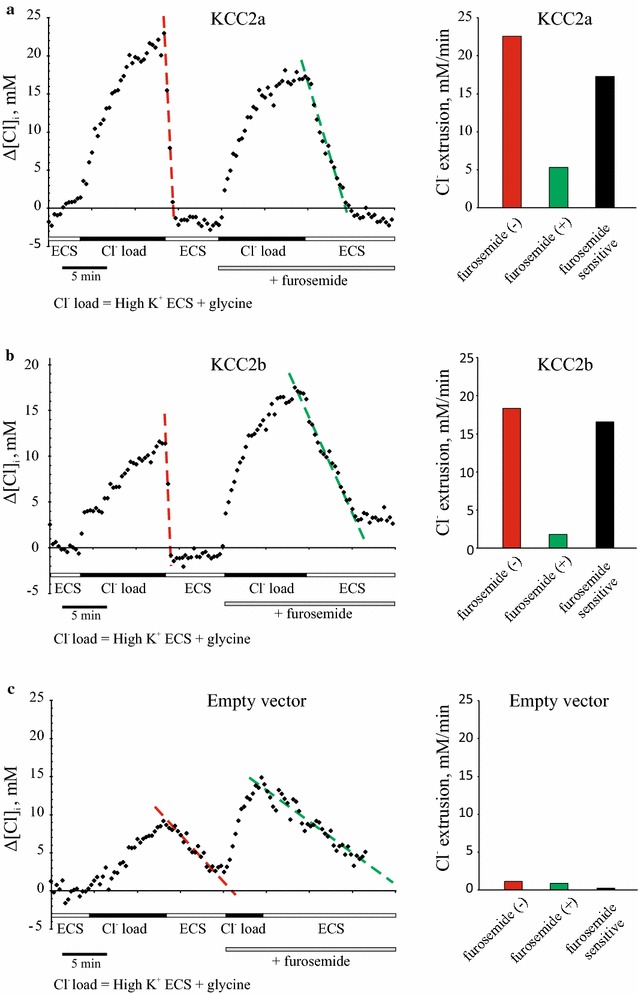
Kinetics of the Cl^−^ extrusion in KCC2a-, KCC2b-, and mock- transfected Neuro-2a cells. Recordings of [Cl^−^]_i_ changes (Δ[Cl^−^]_i_) in Neuro-2a cells transfected with KCC2a (**a**), KCC2b (**b**), and empty vector (**c**) constructs. *Left panels* show individual recordings of representative cells for each of the constructs; *right panels* demonstrate quantifications of the individual recordings. After initial baseline acquisition (4 min), cells were loaded with chloride by applying 100 µM glycine in 50 mM K^+^ ECS (10 min). Washing cells with normal K^+^ ECS for 5–10 min was accompanied by rapid decrease in [Cl^−^]_i_. The rate of the chloride extrusion Δ[Cl^−^]_i_/Δt was much faster in cells transfected with KCC2a (**a**) and KCC2b (**b**) constructs compared to the mock-transfected cells (**c**). In the second phase of the experiment, the whole procedure was repeated in the presence of 500 µM furosemide, a known inhibitor of K–Cl cotransport. The initial rate of [Cl^−^]_i_ reduction (mM/min) was calculated using the first half of each slope. For each individual cell, the rate of furosemide-sensitive Cl extrusion was calculated as a difference between the corresponding rates of [Cl^−^]_i_ extrusion in the presence and absence of furosemide

Mouse neuroblastoma Neuro-2a cells (CCL-131, American Type Culture Collection, Manassas, VA) were cultured in 35 mm culture dishes in Dulbecco’s Modified Eagle’s Medium (DMEM) supplemented with 10% fetal bovine serum and 100 units/ml penicillin plus 100 μg/ml streptomycin antibiotics mix. For imaging experiments, the cells were plated on 1-cm glass coverslips, transfected next day with corresponding DNA constructs using Lipofectamine 2000 (Life Technologies) according to manufacturer’s instructions, and used for Cl^−^ imaging 24–48 h after transfection. The transfection mixes differed in the experiments presented in Figs. 2, 3, and 4, but total DNA amount per one reaction (35 mm plate) was always kept 2.15 µg, of which Cl-sensor constituted 0.25 µg. Since it is important to maintain the same amount of total DNA in all transfection reactions, the empty vector pcDNA3.1(−) was added when needed to adjust the total DNA amount to 2.15 µg. So, for the calibration curve experiments (Fig. 2), the cultures were co-transfected with 0.25 µg of Cl-sensor and 1.9 µg of pcDNA3.1(−). In the experiments intended to optimize the loading procedure (Fig. 3), the transfection mix contained 0.25 µg of the Cl-sensor, 0.7 µg of the human GlyR α1 subunit construct, 0.7 µg of the KCC2 isoform, and 0.5 µg of the pcDNA3.1(−). In the experiments assessing the chloride extrusion activity of the KCC2a and KCC2b isoforms (Fig. 4a, b), the transfection mix contained 0.25 µg of the Cl-sensor, 0.7 µg of the human GlyR α1 subunit expression construct, 0.7 µg of either of the KCC2 isoforms (Fig. 4a, b), and 0.5 µg of the pcDNA3.1(−). For the corresponding control reaction (Fig. 4c, no KCC2), the transfection mix contained 0.25 µg of the Cl-sensor construct, 0.7 µg of the human GlyR α1 subunit expression construct, and 1.2 µg of pcDNA3.1(−). Our approach is based on simultaneous co-expression of multiple constructs that is a reasonable strategy given previous work [56].

### Fluorescence imaging setup

The imaging setup for [Cl^−^]_i_ measurements in neuronal cells transfected with Cl-sensor was based on the inverted Olympus fluorescence microscope (IX71, Olympus, France) and was described previously [57] (Fig. 1b). Briefly, Neuro-2a cells grown on coverslips and expressing Cl-sensor were exited using an X-Cite Series 120Q (Lumen Dynamics Group Inc., Ontario, Canada) light source through the 430(24) nm and 500(20) nm excitation filters (ET430/24× and ET500/20× filters were purchased as a part of the #59217 set, Chroma Technology Corp., Bellows Falls, VT, USA) mounted into the Lambda 10-B Filter wheel (Sutter Instruments Company, Novato, USA). A transmission neutral density filter (ND 1.3 B–5% Trans, Chroma Technology Corp.) was installed in front of 430(24) nm excitation filter to prevent inactivation of the Cl-sensor by 430 nm light [57]. Olympus LUCPlanFLN 20× objective, NA 0.45 (Olympus, France) was used for taking images of 10–20 cells simultaneously (Fig. 1c). The emitted fluorescence passed through a double bandpass [470(24) + 535(30)] filter (59017 m was purchased as a part of the #59217 set, Chroma Technology Corp., Bellows Falls, VT, USA) and was collected using CoolSNAPHQ Monochrome CCD camera with 0.05 Hz acquisition frequency and 20–50 ms exposure time. Intensities of the collected fluorescent signals corresponding to the excitation wavelengths of 430 nm (F_430_) and 500 nm (F_500_) were corrected for background fluorescence. For that, three ROIs were chosen in nonfluorescent areas for each analyzed optic view. Fluorescent intensities measured for three ROIs were averaged (separately for 430 and 500 nm) to obtain the background values that were subsequently subtracted from F_430_ and F_500_ values obtained for ROIs corresponding to the measured cells from the same optic view. Metamorph software with an option for a multi-dimensional acquisition (MDA) (Roper Scientific SAS, Evry, France) was used to calculate a background-corrected ratio R = F_430_/F_500_.

### Fluorescence recording protocol

Coverslips with Neuro-2a cells overexpressing GlyR ɑ1 subunit were fixed inside the imaging chamber, which was mounted onto the inverted Olympus fluorescence microscope. Cells were perfused with an extracellular solution ECS (in mM): 150 NaCl, 2.5 KCl, 2.0 CaCl_2_, 2.0 MgCl_2_, 5 HEPES, 10 d-glucose, pH 7.4 using a fast perfusion system described previously [60]. An inner diameter of the quartz perfusion tubes (250 μm) allowed a rapid (within 100 ms) change of solutions in the entire optical field. Bumetanide (10 μM) was added into all solutions to prevent chloride influx mediated by NKCC1 cotransporter. Chloride loading of Neuro-2a cells was accomplished by applying ECS supplemented with 100 μM glycine. Simultaneously with applying glycine, [K^+^] in the ECS was raised up to 50 mM to increase Cl^−^ driving force and thus to elevate efficiency of Cl^−^ loading process. This was done by substituting [Na^+^] for [K^+^] in the ECS to keep osmolarity of the final solutions equal 310 mOsm. All experiments were performed at room temperature (22–24 °C).

### Calibration

Cl-sensor was calibrated in Neuro-2a cells as described previously [45]. Coverslips were placed in experimental chamber connected to a perfusion system described above. Neuro-2a cells were permeabilized for 5–10 min with 80 mM β-escin (Sigma, St. Louis, USA) added to the extracellular solution (in mM) (150 NaCl, 2.5 KCl, 2.0 CaCl_2_, 2.0 MgCl_2_, 5 HEPES, 10 d-glucose, pH 7.4). Calibration solutions containing different [Cl^−^] (5, 15, 50, 100, and 150 mM) were prepared by mixing solutions with a high chloride concentration (150 mM KCl, 10 mM d-glucose, 20 mM HEPES, pH 7.3) and zero chloride concentration (150 mM K-Gluconate, 10 mM d-glucose, 20 mM HEPES, pH 7.3). The calibration solutions were applied into the experimental chamber through a perfusion system, and intensities of fluorescence F_430_ and F_500_ emitted after excitation of Neuro-2a cells with wavelengths 430 and 500 nm were measured. Incubation time in each of the calibration solutions was about 2–3 min to reach steady-state values for F_430_ and F_500_ signals.

### Statistical analysis

All results are presented as mean ± SEM. The statistical significance was assessed by ANOVA using GraphPad Prizm software (GraphPad Software, CA, USA).

## Results

### Calibration of the Cl-sensor in cultured Neuro-2a cells

We aimed to develop a robust optical technique for measuring K–Cl transport activity mediated by electroneutral K–Cl cotransporter KCC2 in neuronal cells. In contrast to other available optical approaches, we intended to assess chloride fluxes through a plasma membrane rather than a steady-state intracellular chloride concentration [Cl^−^]_i_, as the latter one does not necessarily reflect KCC2 activity properly. Since a regular transport mode of KCC2 is known to be chloride extrusion, we developed our protocol to measure chloride efflux similar to the previously published efflux assays [16, 61, 62]. To visualize [Cl^−^]_i_ dynamics in Neuro-2a cells, we used Cl-sensor, a genetically encoded sensor characterized previously [45, 55, 57]. To calibrate the Cl-sensor for the subsequent flux experiments, we modelled a situation that occurs in Neuro-2a cells, when the chloride flux is generated as a result of the efflux activity of the K–Cl cotransporter. To calibrate Cl-sensor, Neuro-2a cells grown on glass coverslips and expressing Cl-sensor, were permeabilized with β-escin [55] and incubated sequentially in a series of HEPES-buffered solutions with chloride concentrations [Cl^−^]_o_ varying from 5 to 150 mM. Cells were sequentially illuminated by 500 and 430 nm light, and intensities of the fluorescent signals emitted by the Cl-sensor (F_430_ and F_500_), passed through a double bandpass filter [470(24) + 535(30)], were recorded (Fig. 1). The corresponding ratio R = F_430_/F_500_ was calculated for each [Cl^−^]_o_ (Fig. 2a). The changes in the extracellular (and in intracellular due to the β-escin permeabilization) chloride concentrations produced the corresponding changes ΔR. Figure 2a represents an example of one of such experiments, where we applied in series the extracellular solutions with different [Cl^−^]_o_ (50, 15, 5, 50, 100, 150, and 5 mM) to the Neuro-2a cells expressing Cl-sensor and permeabilized with β-escin. Such graphs allowed us to calculate ΔR values for the corresponding Δ[Cl^−^]_o_ changes. Of note, ΔC values can be negative if [Cl^−^]_o_ decreases at the subsequent step, or positive in the opposite situation; similarly, ΔR values can also be positive or negative. Experiments similar to the one shown in the Fig. 2a were performed totally with eight 35 mm dishes (in two independent Neuro-2a cultures), and more than 80 Cl-sensor positive cells were recorded. In these experiments the extracellular solutions with varying [Cl^−^]_o_, were applied in various orders, and the obtained ΔR values were plotted as a function of Δ[Cl^−^]_o_ (Fig. 2b). Nonphysiological Δ[Cl^−^]_o_ values (>100 mM) have not been analyzed. The obtained (Δ[Cl^−^]_o_, ΔR) pairs were fitted by the linear regression with the equation Y = 0.0152*X − 0.0834 (r^2^ = 0.99). In agreement with Stern–Volmer equation, ΔR demonstrated a linear dependence in the broad range of alterations Δ[Cl^−^]_o_ with the slope k = ΔR/Δ[Cl^−^]_o_ = 0.015 (Fig. 2b), though inconsistency in ΔR determination increased for Δ[Cl^−^]_o_ values larger than ± 50 mM. We did not use a nonlinear regression analysis, since the linear regression occurred to be accurate enough for modelling our data in the indicated interval of the chloride changes (<100 mM). In addition, in the case of linear regression, just a single coefficient (k, angle of inclination) is used to transform Δ(F430/F500) into the corresponding changes of the intracellular chloride concentration ΔC.

### Protocol for chloride loading in Neuro-2a cells

One of the strategies for measuring KCC2 activity is to estimate how efficiently KCC2 copes with the artificially elevated [Cl^−^]_i_ level. Chloride loading in such experiments has been successfully accomplished through sharp electrodes [21], patch-clamp glass pipettes [16, 62], and via opening chloride channels of GABA_A_ and Gly receptors upon application of the corresponding agonists [45, 55, 57, 63]. The latter approach allows to change [Cl^−^]_i_ in multiple cells simultaneously, thus it was a method of choice for our experiments. Neuro-2a cells were transiently transfected with the expression construct for GlyR ɑ1 subunit. When expressed in heterologous system, these subunits can form functional homo-oligomeric chloride channels [64] with high Cl^−^ conductance [65]. To enable chloride loading, transfected cells were first pre-incubated in standard ECS for 5 min and then perfused with ECS containing glycine (100 µM) (an arrow in Fig. 3a). ΔR values were recorded every 20 s and transformed into Δ[Cl^−^]_i_ using the slope coefficient k obtained during the calibration procedure. Only minor changes Δ[Cl^−^]_i_ were detected in Neuro-2a cells expressing ɑ1 GlyR upon application of glycine (Fig. 3a). This could be explained by a relatively high basal [Cl^−^]_i_ level (about 60 mM) in Neuro-2a cells [66] and, as a consequence, by a relatively weak inward driving force for chloride. To increase the driving force, we shifted the resting membrane potential (RMP) to more depolarized values simultaneously with the application of glycine (Fig. 3b). For this purpose, Na^+^ in the extracellular solution was substituted for K^+^ to increase concentration of the latter up to 50 mM, while keeping osmolarity constant. Indeed, in high K^+^ conditions we observed a fast and efficient chloride loading in Neuro-2a cells (Fig. 3b). Also, in agreement with previous studies [45, 55, 57], we noticed that high K^+^ itself can induce Cl^−^ loading in Neuro-2a cells even in the absence of glycine application, though a rate of Cl^−^ loading was about twice lower (Fig. 3c). This may be explained by unusually high chloride permeability of Neuro-2a cells reported previously [66] or by the KCl-induced depolarization and subsequent acidification of the intracellular compartments. A combination of high K^+^ and glycine have been used in all subsequent experiments for efficient Cl^−^ loading into Neuro-2a cells.

### Furosemide-sensitive chloride efflux mediated by KCC2a and KCC2b isoforms in Neuro-2a cells

To measure chloride efflux mediated specifically by KCC2, Neuro-2a cells were loaded with Cl^−^ as described above, and kinetics of the subsequent [Cl^−^]_i_ recovery to the baseline level was analyzed sequentially in absence and then in presence of furosemide, a known inhibitor of the K–Cl cotransporters [67]. ΔR values were recorded every 20 s and transformed into changes Δ[Cl^−^]_i_ using the coefficient k derived from calibration procedure (Fig. 2). After 4 min period of baseline recording in normal K^+^ ECS, cells were loaded with Cl^−^ by applying glycine (100 µM) in high K^+^ ECS (Fig. 4a–c). To shorten the imaging protocol, Cl^−^ loading step was restricted to 10 min, even though in most cases this duration was not enough to reach a plateau level. Cl^−^ loading was followed by substitution of the high K^+^ ECS solution for the one with normal K^+^ and containing no glycine that leaded to the fast [Cl^−^]_i_ recovery to the basal level in cells transfected with KCC2a (Fig. 4a) and KCC2b (Fig. 4b), but not with empty vector (Fig. 4c). A linear regression was used to determine a rate of [Cl^−^]_i_ recovery Δ[Cl^−^]_i_/Δt (red lines in Fig. 4a–c), and the corresponding slopes were calculated (red bars on the right panels of Fig. 4a–c). The steep drop of [Cl^−^]_i_ for KCC2a and KCC2b expressing cells can be partly attributed to the KCC2-mediated chloride extrusion and partly to the Cl^−^ efflux via chloride channels and other than KCC2 transporters as a result of the change in the driving force for chloride. Bumetanide (10 µM) was added into all solutions to block the activity of Na^+^–K^+^–2Cl^−^ cotransporter NKCC1, which is known to be endogenously expressed in Neuro-2a cells [66] and also known to mediate chloride influx. In the second part of the recording, the Cl^−^ loading and Cl^−^ recovery steps were repeated in the presence of 500 µM furosemide to block the KCC2-specific efflux activity (green lines and bars in Fig. 4a–c). The furosemide-sensitive components of the Cl^−^ extrusion for individual Neuro-2a cells (black bars in Fig. 4a–c) were derived by subtracting furosemide(+) components from corresponding furosemide(−) components.

**Fig. 5:**
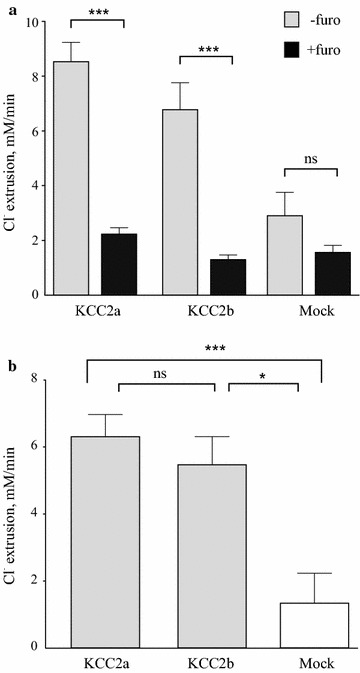
Exogenously expressed KCC2a and KCC2b mediate efficient furosemide-sensitive chloride extrusion in Neuro-2a cells. **a** Mean values for the Cl^−^ extrusion rate in the presence and absence of furosemide obtained in the individual experiments similar to those shown in Fig. 4. The rate of chloride extrusion is significantly decreased by furosemide (500 μM), a known blocker of K–Cl transport, in KCC2a (n = 55) and KCC2b (n = 40) transfected cells. (*** p < 0.005; ns, nonsignificant; two-sided Student’s t-test; *Error bars* represent SEM). **b** Neuro-2a cells expressing KCC2a (n = 55) and KCC2b (n = 40) constructs demonstrate a significantly higher furosemide-sensitive Cl^−^ extrusion rate compared to the mock transfected cells (n = 20). (*p < 0.05; ***p < 0.005; *ns* nonsignificant; Kruskal–Wallis nonparametric ANOVA). Mean values and corresponding SEM are shown

The average chloride efflux characterized by Δ[Cl^−^]_i_/Δt was 2.9-fold higher in cells transfected with KCC2a and 2.3-fold in cells transfected with KCC2b constructs compared to the mock-transfected cells (grey bars in Fig. 5a). Previous studies reported ~2.7-fold increase for ^86^Rb^+^ uptake in HEK293 cells transiently transfected with either KCC2a or KCC2b isoforms [5], and ~fourfold increase in HEK293 stably transfected with KCC2b isoform [67]. Furosemide (500 µM) attenuated Cl^−^ extrusion activity of KCC2a and KCC2b about fourfold, to the level detected in mock-transfected cells in the presence of furosemide (black bars in Fig. 5a). Of note, chloride fluxes in the mock-transfected cells also decreased ~1.9-fold upon furosemide application that is in agreement with ^86^Rb^+^ flux data reported previously for mock-transfected HEK293 cells [5, 67]. Furosemide-sensitive components of Δ[Cl^−^]_i_/Δt values were considerably higher in Neuro-2a cells transfected with KCC2a (6.3-fold) and KCC2b (5.5-fold) constructs compared to the mock-transfected control, but did not differ significantly from each other (Fig. 5b). These values were close to the ones reported previously for the ^86^Rb influx in HEK293 cells stably transfected with KCC2b (6.2-fold) [67], as well as for the ^86^Rb influx in HEK293 cells transiently transfected with KCC2a (~fourfold) and KCC2b (~fourfold) [5, 58].

The above experiments showed that even the relatively short Cl^−^ loading step (~10 min) leads to a significant [Cl^−^]_i_ increase that could affect multiple intracellular signaling pathways. Previous studies have reported an important role of the WNK-SPAK signaling pathway in the regulation of the K–Cl cotransport activity mediated by KCC2: the pathway is known to be fully activated after 30–60 min preincubation in low [Cl^−^]_i_ [68, 69], resulting in KCC2 inhibition [17, 18, 70, 71]. In contrast, the high [Cl^−^]_i_ is known to inhibit SPAK kinase [20], thus leading to the KCC2 activation. Therefore, the Cl^−^ loading step could theoretically lead to the inhibition of the WNK-SPAK pathway and, consequently, to the KCC2 activation, thus affecting the KCC2 transport activity. To address this question, we modified our protocol and included two consecutive chloride-loading steps without the furosemide, and then one loading step with the furosemide. Extrusion kinetics after the first and second chloride-loading steps have been compared for both KCC2a and KCC2b isoforms. For both KCC2 isoforms, we revealed a tendency to the increase of the extrusion rate during the second step compared to the first step, but it was not significant (KCC2a: 61 ± 15%, p = 0.055, Student’s t-test, 47 cells in 3 experiments; KCC2b: 54 ± 25%, p = 0.16, Student’s t-test, 71 cell in 3 experiments). It is important to notice that during our standard protocol (with one chloride-loading step without furosemide and one with furosemide) the tendency to the increase of the extrusion rate cannot distort our measurements, as the KCC2 activity at the second step is anyway blocked by the furosemide.

The 10 min chloride loading protocol was used in our study, since it provided the robust [Cl^−^]_i_ increase for the reasonably short period. The data presented in Fig. 4 also imply that shorter loading protocols can be used to minimize [Cl^−^]_i_ changes and subsequent effects on intracellular signaling pathways (e.g. WNK-SPAK). To check whether the chloride extrusion rate depends on the amount of chloride preloaded during the loading step, we compared the short (2-min) and long (10-min) loading protocols. For the KCC2a-transfected cells in the absence of furosemide, an average increase in the [Cl^−^]_i_ during the 2-min loading step (5.8 ± 0.14 mM, n = 15 cells in N = 4 experiments) was significantly lower (p = 0.0052, Student’s t-test) compared to the 10-min loading protocol (14.1 ± 1.6 mM, n = 57 cells in N = 6 experiments). In the same conditions, the chloride efflux activity after the 2-min loading procedure (7.3 ± 0.24 mM/min, n = 15 cells in N = 4 experiments) was not significantly different (p = 0.19, Student’s t-test) compared to the efflux activity after the 10-min loading step (8.4 ± 1.6 mM/min, n = 57 cells in N = 6 experiments). For the KCC2a-transfected cells in the presence of furosemide, an average increase in the [Cl^−^]_i_ for the 2-min loading step (6.9 ± 0.37 mM, n = 25 cells in N = 4 experiments) was significantly lower (p = 0.035, Student’s t-test) compared to the 10-min loading (13.4 ± 2.1 mM, n = 51 cell in N = 6 experiments). However, the chloride efflux activity after the 2-min loading procedure (2.6 ± 0.36 mM/min, n = 15 cells in N = 4 experiments) was not significantly different (p = 0.59, Student’s t-test) compared to the efflux activity after the 10-min loading step (2.4 ± 0.6 mM/min, n = 51 cells in N = 6 experiments). Importantly, in both cases (2-min and 10-min loading) we did not observe significant difference for amount of chloride loaded in the presence and absence of furosemide. For the 2-min loading step: (−furo) 5.8 ± 0.14 mM, n = 15 cells in N = 4 experiments versus (+furo) 6.9 ± 0.37 mM, n = 25 cells in N = 4 experiments, p = 0.07, Student’s t-test. For the 10-min loading step: (−furo) 14.1 ± 1.6 mM, n = 57 cells in N = 6 experiments versus (+furo) 13.4 ± 2.1 mM, n = 51 cell in N = 6 experiments, p = 0.8, Student’s t-test. Thus, we conclude that the efflux activity of the KCC2a cotransporter does not depend on the amount of chloride pre-loaded into the cells. In addition, these data show that furosemide does not affect significantly the amount of chloride loaded during the loading step.

## Discussion

In this study we have described a straightforward technique for assessing efflux activity mediated by K–Cl cotransporter KCC2 in neuronal cells. Our protocol ensures following features: (1) physiologically relevant chloride sensitivity, (2) direct assessment of furosemide-sensitive chloride fluxes instead of a steady-state chloride concentration, (3) measurement of chloride efflux instead of influx, (4) minimal impact of intracellular signaling pathways (e.g. SPAK/OSR1), (5) noninvasiveness, and (6) high throughput. While some of these features individually can be attributed to currently known methods for assessing KCC2 activity, none of the existing methods to our knowledge has so far combined all these features.

As a basis for our technique, we have chosen an optical approach and implemented it by means of the previously characterized Cl-sensor, a triple YFP mutant H148Q/I152L/V163S [45]. Optical approaches in general are known to provide low toxicity, noninvasiveness, and high efficiency. Although expression level of Cl-sensor in our experiments was high enough for stable recordings of chloride fluxes, no signs of cytotoxicity were observed. Morphology of Neuro-2a cells expressing Cl-sensor was normal and did not differ from that of non-transfected cells. Cl-sensor in combination with our imaging setup, which is equipped with 20× objective, allowed us to measure simultaneously the furosemide-sensitive chloride fluxes in about 15 transfected cells during our standard (about 40 min) recording protocol. Similar throughput has been previously observed in studies that used Cl-sensor in dissociated cultures [45, 55, 57], brain and retina slices [55], and ex vivo whole-mount DRG samples [49]. More cells can be analyzed by using objectives with lower magnification, but this may require increasing an acquisition time and/or intensity of light sources. Both these factors affect accuracy of Cl^−^ measurements [57], partially because YFP and CFP components of Cl-sensor have differential photobleaching rates [51, 72]. Thus, to prevent photobleaching we kept acquisition time (20–50 ms) and rate (0.05 Hz) minimal. For cells with a very high KCC2 activity, time required for restoration of [Cl^−^]_i_ to the basal level may become too short, thus temporal resolution of the acquisition process may need to be increased. Intrinsic properties of the YFP mutant utilized for Cl-sensor design impose an upper limit for an acquisition rate of about 0.5 Hz [38], thus temporal resolution can be further increased.

Development of GECS has been significantly intensified during last decade, and different chloride sensors have been used for measuring steady-state [Cl^−^]_i_ levels in mammalian cells [35]. However, it has previously been noticed that the steady-state [Cl^−^]_i_ level itself does not necessarily reflect KCC2 extrusion activity properly, as in the conditions of low chloride conductance even a relatively weak KCC2 activity may significantly decrease [Cl^−^]_i_ [16, 62]. Thus, we used Cl-sensor to assess KCC2-mediated chloride efflux by measuring directly Δ[Cl^−^]_i_ in time. This approach required a different calibration protocol for Cl-sensor: while a standard calibration establishes relationship between absolute values of F_430_/F_500_ ratio and [Cl^−^]_i_, our protocol determines a link between changes ΔR of the F_430_/F_500_ ratio and changes Δ[Cl^−^]_i_ (Fig. 2b). Changes in [Cl^−^]_i_ were induced by applying calibration solutions with predefined chloride concentrations [Cl^−^]_o_ to Neuro-2a cells permeabilized with β-escin. Importantly, ΔR and Δ[Cl^−^]_i_ were linearly related that is in agreement with previous studies [73], although inconsistency in ΔR determination increased outside physiological range for Δ[Cl^−^]_i_ values larger than ± 50 mM (Fig. 2b). The angle of inclination between the regression line and x-axis allowed us to estimate dissociation constant K_d_ defined according to the Stern–Volmer equation as R_0_/K_d_, where R_0_ is F_430_/F_500_ ratio at zero chloride concentration. Dissociation constant for the Cl-sensor in Neuro-2a cells was found to be ~60 mM that is close to the previously calculated K_d_ ~ 50 mM for Cl-sensor in cultured neurons [55] as well as for other double and triple YFP mutants analogous to Cl-sensor [38]: H148Q/I152L (~90 mM), H148Q/V150A/I152L (~60 mM), and H148Q/V163S (~60 mM). The calculated K_d_ ~ 60 mM is close to the value of the basal [Cl^−^]_i_ level in Neuro-2a cells [66], thus making Cl-sensor suitable for assessing chloride fluxes in this model system.

Our protocol includes Cl^−^ loading in Neuro-2a cells with subsequent analysis of how fast KCC2 brings down [Cl^−^]_i_ to the basal level. Previous studies showed that certain GluR subunits form functional homo-oligomeric glycine receptors possessing high Cl^−^ conductance. We used ɑ1 subunit of GlyR to load Neuro-2a cells with Cl^−^ upon application of glycine. Preliminary experiments showed that Cl^−^ loading was relatively weak that could be explained by a relatively high basal [Cl^−^]_i_ in Neuro-2a cells. To make the loading process more efficient, in parallel with glycine application we elevated resting membrane potential by substituting extracellular Na^+^ for K^+^ in ECS medium (high K^+^ medium). The K^+^-induced depolarization is known to result in [Ca^2+^]_i_ increase that in turn, via Ca^2+^-ATPases, could result in acidification up to 0.2 pH units [74]. Since all YFP-based chloride sensors are known to be pH sensitive, the K^+^-induced depolarization may affect the Cl-sensor recordings. By using Cl-free solutions, the pH-associated Δ[Cl^−^]_i_ values, reported by Cl-sensor, were estimated to be ~5 mM for the 4 min acidification interval [45]. Slightly higher Δ[Cl^−^]_i_ values (~7 mM) have been observed in our system for the same 4 min acidification interval (Fig. 3c). In contrast to the Markova and coauthors, we did not substitute chloride by gluconate in our solutions, thus the slightly increased Δ[Cl^−^]_i_ values could be attributed, for example, to the increased Cl-influx activity of the chloride channels/transporters in the Neuro-2a cells upon the increased driving force induced by KCl application. This approach resulted in a robust Cl^−^ load, increasing intracellular concentration by ~15–20 mM in Neuro-2a cells expressing KCC2 constructs and ~10–15 mM in the mock transfected cells (Fig. 4). Importantly, amplitude of [Cl^−^]_i_ changes during the Cl^−^ loading procedure did not exceed the ±50 mM range of the standard curve (Fig. 2b) that represents a linear interval of dependence between ΔR and Δ[Cl^−^]_i_. Higher efficiencies of the Cl^−^ loading in KCC2-expressing cells compared to the mock-transfected cells could be explained by the inverted mode of KCC2-mediated transport in the conditions of elevated K^+^, as it was predicted previously [67]. Even though the inverted mode (influx) is commonly used to assess K–Cl cotransport activity mediated by KCC2 and other members of CCC family, we are not aware of any studies demonstrating that these two approaches are equivalent. Thus, we optimized our approach according to previous studies that measured KCC mediated chloride efflux [61, 62, 75, 76].

The idea of our approach has been borrowed from another method for assessing the chloride extrusion/intrusion activity—rubidium assay, which is widely used in the chloride-cotransporter field for decades. According to the methodology of the rubidium assay, a conclusion about the transporter activity can be made on the bases of two measurements: (1) in normal conditions, and (2) in the same conditions, but in the presence of the inhibitor for the transporter of interest. It is important to note that the rubidium flux through a plasma membrane is mediated not only by the activity of the transporter of interest, but also through dozens of other channels and transporters endogenously expressed in the model system. Therefore, an estimation of individual impacts of each of these channels/transporters would be extremely laborious and complex. Instead, a transport activity of a protein of interest is calculated by subtracting the transport activities in the absence and in the presence of the inhibitor. The same approach is used in our method, and it is the main dignity of our method that it eliminates necessity to meticulously calibrate Cl-sensor dependence on numerous parameters, some of which possibly have not been even recognized yet. Indeed, we are still not aware about all characteristics of the Cl-sensor and its dependence on any other factors except pH. We also do not know how other biologically significant intracellular components and cascades affect the Cl-sensor. That is why we proposed to assess chloride fluxes using the Cl-sensor first in standard conditions and immediately after that in the presence of furosemide, a well-known KCC2 inhibitor.

Changing ECS medium to the one with normal K^+^ and without glycine resulted in a rapid chloride efflux via KCC2-dependent and, possibly, via some KCC2-independent mechanisms. The latter component, represented by chloride channels and transporters other than KCC2, was measured by repeating the experiment in the presence of furosemide, a known inhibitor of K–Cl cotransporters. The furosemide(+) component of the efflux was relatively high (Fig. 5a), thus confirming a previously known high chloride permeability of Neuro-2a cells [66]. Average values of the furosemide(+) components measured in KCC2a, KCC2b, and mock transfected cells were comparable (Fig. 5a), indicating that 500 µM furosemide blocked efficiently both KCC2a and KCC2b mediated chloride transport. Furosemide concentrations up to 2 mM were used previously to block activity of KCC2 and other K–Cl cotransporters in cell lines and oocytes [5, 32, 57, 77–80], despite the fact that original studies in HEK293 cells identified K_i_ for furosemide to be around 25 µM for KCC2 [67] and 40 µM for KCC1 [61]. Even though the inhibitory effect of furosemide in the mock-transfected cells was not significant, a clear tendency towards downregulation of the chloride extrusion rate was observed (Fig. 5a) indicating that endogenous expression of K–Cl cotransporters in Neuro-2a cells cannot be excluded. Indeed, we have previously reported a weak endogenous expression of KCC2 in Neuro-2a cells [81] that could be further increased upon differentiation of Neuro-2a cells by retinoic acid (data not shown). A similar inhibitory effect of furosemide in mock-transfected cells has been reported for HEK293 cells [5, 67, 79] in agreement with data showing endogenous KCC1 and KCC4 expression in HEK293 cells [82]. We found that the furosemide-sensitive chloride efflux was considerably higher in Neuro-2a cells transfected with KCC2a (6.3-fold) and KCC2b (5.5-fold) constructs compared to the mock-transfected control, but did not differ significantly from each other (Fig. 5b). These values are close to the ones reported previously for the ^86^Rb influx (6.2-fold) in HEK293 cells stably transfected with KCC2b [67] as well as for the ^86^Rb influx in HEK293 cells transiently transfected with KCC2a (~fourfold) and KCC2b (~fourfold) [5].

## Conclusions

In this study we presented a robust and reliable approach for measuring K–Cl transport activity mediated by neuronal cotransporter KCC2. Our noninvasive approach proved to be efficient for measuring physiologically relevant chloride concentrations, allowed direct assessment of furosemide-sensitive chloride fluxes, and in contrast to many existing techniques allowed to assess chloride efflux instead of influx. We used this method to compare transport activities of the N-terminal splice isoforms KCC2a and KCC2b in neuronal cells. Results obtained with our approach matched well to the results acquired previously using standard methods.

## Abbreviations

CCC: cation-chloride cotransporter
KCC2: K–Cl cotransporter member 2
GABA: γ-aminobutyric acid
HTS: high-throughput screening
GABA_A_: inotropic γ-aminobutyric acid type A receptors
GlyR: glycine receptors
E_GABA_: reversal potential for inotropic γ-aminobutyric acid type A receptors
E_Gly_: reversal potential for inotropic glycine receptors
SPAK: STE20/SPS1-related proline-alanine-rich protein kinase
OSR1: oxidative-stress responsive protein kinase 1
IPSP_A_: inhibitory postsynaptic potential mediated by γ-aminobutyric acid type A receptors
GECS: genetically encoded chloride sensors
GFP: green fluorescent protein
YFP: yellow fluorescent protein
FRET: Förster resonance energy transfer
DMEM: Dulbecco’s Modified Eagle’s Medium
ECS: extracellular solution
NKCC1: sodium (Na+) potassium (K+) chloride (Cl−) cotransporter member 1

## References

[1] Gamba G (2005). Molecular physiology and pathophysiology of electroneutral cation-chloride cotransporters. Physiol Rev.

[2] Payne JA, Stevenson TJ, Donaldson LF (1996). Molecular characterization of a putative K–Cl cotransporter in rat brain. A neuronal-specific isoform. J Biol Chem..

[3] Kaila K, Price TJ, Payne JA, Puskarjov M, Voipio J (2014). Cation-chloride cotransporters in neuronal development, plasticity and disease. Nat Rev Neurosci.

[4] Rivera C, Voipio J, Payne JA, Ruusuvuori E, Lahtinen H, Lamsa K, Pirvola U, Saarma M, Kaila K (1999). The K +/Cl- co-transporter KCC2 renders GABA hyperpolarizing during neuronal maturation. Nature.

[5] Uvarov P, Ludwig A, Markkanen M, Pruunsild P, Kaila K, Delpire E, Timmusk T, Rivera C, Airaksinen MS (2007). A novel N-terminal isoform of the neuron-specific K–Cl cotransporter KCC2. J Biol Chem.

[6] Hubner CA, Stein V, Hermans-Borgmeyer I, Meyer T, Ballanyi K, Jentsch TJ (2001). Disruption of KCC2 reveals an essential role of K–Cl cotransport already in early synaptic inhibition. Neuron.

[7] Tornberg J, Voikar V, Savilahti H, Rauvala H, Airaksinen MS (2005). Behavioural phenotypes of hypomorphic KCC2-deficient mice. Eur J Neurosci.

[8] Woo NS, Lu J, England R, McClellan R, Dufour S, Mount DB, Deutch AY, Lovinger DM, Delpire E (2002). Hyperexcitability and epilepsy associated with disruption of the mouse neuronal-specific K–Cl cotransporter gene. Hippocampus..

[9] Puskarjov M, Seja P, Heron SE, Williams TC, Ahmad F, Iona X, Oliver KL, Grinton BE, Vutskits L, Scheffer IE, Petrou S, Blaesse P, Dibbens LM, Berkovic SF, Kaila K (2014). A variant of KCC2 from patients with febrile seizures impairs neuronal Cl- extrusion and dendritic spine formation. EMBO Rep.

[10] Kahle KT, Merner ND, Friedel P, Silayeva L, Liang B, Khanna A, Shang Y, Lachance-Touchette P, Bourassa C, Levert A, Dion PA, Walcott B, Spiegelman D, Dionne-Laporte A, Hodgkinson A, Awadalla P, Nikbakht H, Majewski J, Cossette P, Deeb TZ, Moss SJ, Medina I, Rouleau GA (2014). Genetically encoded impairment of neuronal KCC2 cotransporter function in human idiopathic generalized epilepsy. EMBO Rep.

[11] Stodberg T, McTague A, Ruiz AJ, Hirata H, Zhen J, Long P, Farabella I, Meyer E, Kawahara A, Vassallo G, Stivaros SM, Bjursell MK, Stranneheim H, Tigerschiold S, Persson B, Bangash I, Das K, Hughes D, Lesko N, Lundeberg J, Scott RC, Poduri A, Scheffer IE, Smith H, Gissen P, Schorge S, Reith ME, Topf M, Kullmann DM, Harvey RJ, Wedell A, Kurian MA (2015). Mutations in SLC12A5 in epilepsy of infancy with migrating focal seizures. Nat Commun..

[12] Coull JA, Boudreau D, Bachand K, Prescott SA, Nault F, Sik A, De Koninck P, De Koninck Y (2003). Trans-synaptic shift in anion gradient in spinal lamina I neurons as a mechanism of neuropathic pain. Nature.

[13] Gagnon M, Bergeron MJ, Lavertu G, Castonguay A, Tripathy S, Bonin RP, Perez-Sanchez J, Boudreau D, Wang B, Dumas L, Valade I, Bachand K, Jacob-Wagner M, Tardif C, Kianicka I, Isenring P, Attardo G, Coull JA, De Koninck Y (2013). Chloride extrusion enhancers as novel therapeutics for neurological diseases. Nat Med.

[14] Kahle KT, Khanna A, Clapham DE, Woolf CJ (2014). Therapeutic restoration of spinal inhibition via druggable enhancement of potassium-chloride cotransporter KCC2-mediated chloride extrusion in peripheral neuropathic pain. JAMA Neurol..

[15] Ebihara S, Shirato K, Harata N, Akaike N (1995). Gramicidin-perforated patch recording: GABA response in mammalian neurones with intact intracellular chloride. J Physiol.

[16] Jarolimek W, Lewen A, Misgeld U (1999). A furosemide-sensitive K + -Cl- cotransporter counteracts intracellular Cl- accumulation and depletion in cultured rat midbrain neurons. J Neurosci.

[17] Inoue K, Furukawa T, Kumada T, Yamada J, Wang T, Inoue R, Fukuda A (2012). Taurine inhibits K + -Cl- cotransporter KCC2 to regulate embryonic Cl- homeostasis via with-no-lysine (WNK) protein kinase signaling pathway. J Biol Chem.

[18] de Los Heros P, Alessi DR, Gourlay R, Campbell DG, Deak M, Macartney TJ, Kahle KT, Zhang J (2014). The WNK-regulated SPAK/OSR1 kinases directly phosphorylate and inhibit the K + -Cl- co-transporters. Biochem J.

[19] Kahle KT, Khanna AR, Alper SL, Adragna NC, Lauf PK, Sun D, Delpire E (2015). K–Cl cotransporters, cell volume homeostasis, and neurological disease. Trends Mol Med..

[20] Gagnon KB, England R, Delpire E (2006). Characterization of SPAK and OSR1, regulatory kinases of the Na-K-2Cl cotransporter. Mol Cell Biol.

[21] Thompson SM, Deisz RA, Prince DA (1988). Outward chloride/cation co-transport in mammalian cortical neurons. Neurosci Lett.

[22] Deisz RA, Lehmann TN, Horn P, Dehnicke C, Nitsch R (2011). Components of neuronal chloride transport in rat and human neocortex. J Physiol.

[23] Deisz RA, Wierschke S, Schneider UC, Dehnicke C (2014). Effects of VU0240551, a novel KCC2 antagonist, and DIDS on chloride homeostasis of neocortical neurons from rats and humans. Neuroscience.

[24] Verkman AS, Galietta LJ (2009). Chloride channels as drug targets. Nat Rev Drug Discov..

[25] Norez C, Heda GD, Jensen T, Kogan I, Hughes LK, Auzanneau C, Derand R, Bulteau-Pignoux L, Li C, Ramjeesingh M, Li H, Sheppard DN, Bear CE, Riordan JR, Becq F (2004). Determination of CFTR chloride channel activity and pharmacology using radiotracer flux methods. J Cyst Fibros.

[26] Qi J, Wang Y, Liu Y, Zhang F, Guan B, Zhang H (2014). Development and validation of HTS assay for screening the calcium-activated chloride channel modulators in TMEM16A stably expressed CHO cells. Anal Bioanal Chem.

[27] Bartschat DK, Blaustein MP (1985). Calcium-activated potassium channels in isolated presynaptic nerve terminals from rat brain. J Physiol.

[28] Bartschat DK, Blaustein MP (1985). Potassium channels in isolated presynaptic nerve terminals from rat brain. J Physiol.

[29] Tang W, Kang J, Wu X, Rampe D, Wang L, Shen H, Li Z, Dunnington D, Garyantes T (2001). Development and evaluation of high throughput functional assay methods for HERG potassium channel. J Biomol Screen.

[30] Scott CW, Wilkins DE, Trivedi S, Crankshaw DJ (2003). A medium-throughput functional assay of KCNQ2 potassium channels using rubidium efflux and atomic absorption spectrometry. Anal Biochem.

[31] Weaver CD, Harden D, Dworetzky SI, Robertson B, Knox RJ (2004). A thallium-sensitive, fluorescence-based assay for detecting and characterizing potassium channel modulators in mammalian cells. J Biomol Screen.

[32] Zhang D, Gopalakrishnan SM, Freiberg G, Surowy CS (2010). A thallium transport FLIPR-based assay for the identification of KCC2-positive modulators. J Biomol Screen.

[33] Carmosino M, Rizzo F, Torretta S, Procino G, Svelto M (2013). High-throughput fluorescent-based NKCC functional assay in adherent epithelial cells. BMC Cell Biol..

[34] Liu X, Titz S, Lewen A, Misgeld U (2003). KCC2 mediates NH4 + uptake in cultured rat brain neurons. J Neurophysiol.

[35] Arosio D, Ratto GM (2014). Twenty years of fluorescence imaging of intracellular chloride. Front Cell Neurosci.

[36] Wachter RM, Remington SJ (1999). Sensitivity of the yellow variant of green fluorescent protein to halides and nitrate. Curr Biol.

[37] Jayaraman S, Haggie P, Wachter RM, Remington SJ, Verkman AS (2000). Mechanism and cellular applications of a green fluorescent protein-based halide sensor. J Biol Chem.

[38] Galietta LJ, Haggie PM, Verkman AS (2001). Green fluorescent protein-based halide indicators with improved chloride and iodide affinities. FEBS Lett.

[39] Watts SD, Suchland KL, Amara SG, Ingram SL (2012). A sensitive membrane-targeted biosensor for monitoring changes in intracellular chloride in neuronal processes. PLoS ONE.

[40] Kuner T, Augustine GJ (2000). A genetically encoded ratiometric indicator for chloride: capturing chloride transients in cultured hippocampal neurons. Neuron.

[41] Grimley JS, Li L, Wang W, Wen L, Beese LS, Hellinga HW, Augustine GJ (2013). Visualization of synaptic inhibition with an optogenetic sensor developed by cell-free protein engineering automation. J Neurosci.

[42] Arosio D, Ricci F, Marchetti L, Gualdani R, Albertazzi L, Beltram F (2010). Simultaneous intracellular chloride and pH measurements using a GFP-based sensor. Nat Methods.

[43] Mukhtarov M, Liguori L, Waseem T, Rocca F, Buldakova S, Arosio D, Bregestovski P (2013). Calibration and functional analysis of three genetically encoded Cl(−)/pH sensors. Front Mol Neurosci.

[44] Raimondo JV, Joyce B, Kay L, Schlagheck T, Newey SE, Srinivas S, Akerman CJ (2013). A genetically-encoded chloride and pH sensor for dissociating ion dynamics in the nervous system. Front Cell Neurosci.

[45] Markova O, Mukhtarov M, Real E, Jacob Y, Bregestovski P (2008). Genetically encoded chloride indicator with improved sensitivity. J Neurosci Methods.

[46] Mukhtarov M, Markova O, Real E, Jacob Y, Buldakova S, Bregestovski P (1880). Monitoring of chloride and activity of glycine receptor channels using genetically encoded fluorescent sensors. Philos Trans A Math Phys Eng Sci.

[47] Metzger F, Repunte-Canonigo V, Matsushita S, Akemann W, Diez-Garcia J, Ho CS, Iwasato T, Grandes P, Itohara S, Joho RH, Knopfel T (2002). Transgenic mice expressing a pH and Cl- sensing yellow-fluorescent protein under the control of a potassium channel promoter. Eur J Neurosci.

[48] Berglund K, Schleich W, Krieger P, Loo LS, Wang D, Cant NB, Feng G, Augustine GJ, Kuner T (2006). Imaging synaptic inhibition in transgenic mice expressing the chloride indicator. Clomeleon. Brain Cell Biol.

[49] Batti L, Mukhtarov M, Audero E, Ivanov A, Paolicelli RC, Zurborg S, Gross C, Bregestovski P, Heppenstall PA (2013). Transgenic mouse lines for non-invasive ratiometric monitoring of intracellular chloride. Front Mol Neurosci.

[50] Elsliger MA, Wachter RM, Hanson GT, Kallio K, Remington SJ (1999). Structural and spectral response of green fluorescent protein variants to changes in pH. Biochemistry.

[51] Bregestovski P, Waseem T, Mukhtarov M (2009). Genetically encoded optical sensors for monitoring of intracellular chloride and chloride-selective channel activity. Front Mol Neurosci.

[52] Berglund K, Kuner T, Feng G, Augustine GJ (2011). Imaging synaptic inhibition with the genetically encoded chloride indicator Clomeleon. Cold Spring Harb Protoc.

[53] Chesler M (2003). Regulation and modulation of pH in the brain. Physiol Rev.

[54] Chesler M, Kaila K (1992). Modulation of pH by neuronal activity. Trends Neurosci.

[55] Waseem T, Mukhtarov M, Buldakova S, Medina I, Bregestovski P (2010). Genetically encoded Cl-sensor as a tool for monitoring of Cl-dependent processes in small neuronal compartments. J Neurosci Methods.

[56] Pellegrino C, Gubkina O, Schaefer M, Becq H, Ludwig A, Mukhtarov M, Chudotvorova I, Corby S, Salyha Y, Salozhin S, Bregestovski P, Medina I (2011). Knocking down of the KCC2 in rat hippocampal neurons increases intracellular chloride concentration and compromises neuronal survival. J Physiol.

[57] Friedel P, Bregestovski P, Medina I (2013). Improved method for efficient imaging of intracellular Cl(−) with Cl-sensor using conventional fluorescence setup. Front Mol Neurosci.

[58] Uvarov P, Ludwig A, Markkanen M, Soni S, Hubner CA, Rivera C, Airaksinen MS (2009). Coexpression and heteromerization of two neuronal K–Cl cotransporter isoforms in neonatal brain. J Biol Chem.

[59] De Saint Jan D, David-Watine B, Korn H, Bregestovski P (2001). Activation of human alpha1 and alpha2 homomeric glycine receptors by taurine and GABA. J Physiol.

[60] Medina I, Filippova N, Barbin G, Ben-Ari Y, Bregestovski P (1994). Kainate-induced inactivation of NMDA currents via an elevation of intracellular Ca2 + in hippocampal neurons. J Neurophysiol.

[61] Gillen CM, Brill S, Payne JA, Forbush B (1996). III. Molecular cloning and functional expression of the K–Cl cotransporter from rabbit, rat, and human. A new member of the cation-chloride cotransporter family. J Biol Chem.

[62] Khirug S, Huttu K, Ludwig A, Smirnov S, Voipio J, Rivera C, Kaila K, Khiroug L (2005). Distinct properties of functional KCC2 expression in immature mouse hippocampal neurons in culture and in acute slices. Eur J Neurosci.

[63] Inoue K, Yamada J, Ueno S, Fukuda A (2006). Brain-type creatine kinase activates neuron-specific K + -Cl- co-transporter KCC2. J Neurochem.

[64] Pribilla I, Takagi T, Langosch D, Bormann J, Betz H (1992). The atypical M2 segment of the beta subunit confers picrotoxinin resistance to inhibitory glycine receptor channels. EMBO J.

[65] Bormann J, Rundstrom N, Betz H, Langosch D (1993). Residues within transmembrane segment M2 determine chloride conductance of glycine receptor homo- and hetero-oligomers. EMBO J.

[66] Bettendorff L, Lakaye B, Margineanu I, Grisar T, Wins P (2002). ATP-driven, Na(+)-independent inward Cl − pumping in neuroblastoma cells. J Neurochem.

[67] Payne JA (1997). Functional characterization of the neuronal-specific K–Cl cotransporter: implications for [K^+^]_o_ regulation. Am J Physiol.

[68] Dowd BFX, Forbush B (2003). PASK (proline-alanine-rich STE20-related kinase), a regulatory kinase of the Na–K–Cl cotransporter (NKCC1) 1. J Biol Chem.

[69] Flemmer AW, Gimenez I, Dowd BF, Darman RB, Forbush B (2002). Activation of the Na–K–Cl cotransporter NKCC1 detected with a phospho-specific antibody. J Biol Chem.

[70] Friedel P, Kahle KT, Zhang J, Hertz N, Pisella LI, Buhler E, Schaller F, Duan J, Khanna AR, Bishop PN, Shokat KM, Medina I (2015). WNK1-regulated inhibitory phosphorylation of the KCC2 cotransporter maintains the depolarizing action of GABA in immature neurons. Sci Signal.

[71] Kahle KT, Delpire E (2016). Kinase-KCC2 coupling: Cl − rheostasis, disease susceptibility, therapeutic target. J Neurophysiol.

[72] Tramier M, Zahid M, Mevel JC, Masse MJ, Coppey-Moisan M (2006). Sensitivity of CFP/YFP and GFP/mCherry pairs to donor photobleaching on FRET determination by fluorescence lifetime imaging microscopy in living cells. Microsc Res Tech.

[73] Arosio D, Garau G, Ricci F, Marchetti L, Bizzarri R, Nifosi R, Beltram F (2007). Spectroscopic and structural study of proton and halide ion cooperative binding to gfp. Biophys J.

[74] Slemmer JE, Matsushita S, De Zeeuw CI, Weber JT, Knopfel T (2004). Glutamate-induced elevations in intracellular chloride concentration in hippocampal cell cultures derived from EYFP-expressing mice. Eur J Neurosci.

[75] Rust MB, Alper SL, Rudhard Y, Shmukler BE, Vicente R, Brugnara C, Trudel M, Jentsch TJ, Hubner CA (2007). Disruption of erythroid K–Cl cotransporters alters erythrocyte volume and partially rescues erythrocyte dehydration in SAD mice. J Clin Invest.

[76] Shen MR, Chou CY, Hsu KF, Liu HS, Dunham PB, Holtzman EJ, Ellory JC (2001). The KCl cotransporter isoform KCC3 can play an important role in cell growth regulation. Proc Natl Acad Sci USA.

[77] Strange K, Singer TD, Morrison R, Delpire E (2000). Dependence of KCC2 K–Cl cotransporter activity on a conserved carboxy terminus tyrosine residue. Am J Physiol Cell Physiol.

[78] Delpire E, Days E, Lewis LM, Mi D, Kim K, Lindsley CW, Weaver CD (2009). Small-molecule screen identifies inhibitors of the neuronal K–Cl cotransporter KCC2. Proc Natl Acad Sci USA.

[79] Hartmann AM, Nothwang HG (2011). Opposite temperature effect on transport activity of KCC2/KCC4 and N(K)CCs in HEK-293 cells. BMC Res Notes.

[80] Mount DB, Mercado A, Song L, Xu J, George AL, Delpire E, Gamba G (1999). Cloning and characterization of KCC3 and KCC4, new members of the cation-chloride cotransporter gene family. J Biol Chem.

[81] Uvarov P, Ludwig A, Markkanen M, Rivera C, Airaksinen MS (2006). Upregulation of the neuron-specific K +/Cl − cotransporter expression by transcription factor early growth response 4. J Neurosci.

[82] Simard CF, Bergeron MJ, Frenette-Cotton R, Carpentier GA, Pelchat ME, Caron L, Isenring P (2007). Homooligomeric and heterooligomeric associations between K + –Cl − cotransporter isoforms and between K + –Cl − and Na + –K + –Cl − cotransporters. J Biol Chem.

